# Prevalence of cytopenias in both HAART and HAART naïve HIV infected adult patients in Ethiopia: a cross sectional study

**DOI:** 10.1186/s12878-018-0102-7

**Published:** 2018-04-05

**Authors:** Tamirat Edie Fekene, Leja Hamza Juhar, Chernet Hailu Mengesha, Dawit Kibru Worku

**Affiliations:** 1 Department of internal medicine, College of Public Health and Medical Sciences, Jimma University, P.O. Box376, Jimma, Ethiopia; 2 Department of Epidemiology, Jimma University, P.O. Box 376, Jimma, Ethiopia; 3 Department of Internal Medicine, Bahir Dar University, -79 Bahir Dar, Ethiopia

**Keywords:** Anemia, Leucopenia, Lymphopenia, Thrombocytopenia, HIV, HAART, CPT

## Abstract

**Background:**

In individuals infected with HIV, hematological abnormalities are common and are associated with increased risk of disease progression and death. However, the profile of hematological abnormalities in HIV infected adult patients is not known in Ethiopia. Thus, the aim of this study was to assess the hematological manifestations of HIV infection and to identify the factors associated with cytopenias in both HAART and HAART naïve HIV infected adult patients in Ethiopia.

**Method:**

We conducted a cross-sectional quantitative study of HIV-infected adult patients attending the ART follow-up clinic of Jimma University Specialized Hospital in Jimma, Ethiopia, from July 2012 to September 2012. We used a structured questionnaire to collect socio-demographic and clinical information. After interviewing, 4 ml of venous blood was drawn from each study subject for hematologic and immunologic parameters.

**Result:**

The prevalence of anemia, leucopenia, thrombocytopenia and lymphopenia among the study individuals were 51.5%, 13%, 11.1% and 5% respectively. Presence of opportunistic infection (*p* = 0.001), use of CPT (*p* = 0.04) and CD4 count < 200 cells/μl (*p* = 0.002) were associated with an increased risk of anemia.

**Conclusion:**

Hematologic abnormalities were common in HIV infected adult patients. Of the cytopenias anemia was the most common. Use of CPT was independently associated with increased risk of anemia and leucopenia. Therefore, large scale and longitudinal studies, giving emphasis on the association of CPT and cytopenia, are recommended to strengthen and explore the problem in depth.

## Background

In 2008, an estimated 33.4 million people were living with HIV/AIDS worldwide; nearly 70% of these were found in sub-Saharan Africa. Since the beginning of the epidemic, almost 60 million people have been infected with HIV and 25 million people have died of HIV-related causes. [1].

Altered hematopoiesis (blood cell production) occurs in patients with HIV infection. This change affects all three cell lines (red blood cells, white blood cells, and platelets) and consequently, HIV-infected patients may suffer from anemia, leucopenia, thrombocytopenia, or any combination of these three. They are common throughout the course of HIV infection and may be the direct result of HIV; at the stem cell level or the mature blood cell level; manifestations of secondary infections and neoplasms, or side effects of therapy. The use of cART could positively or negatively affect these parameters, depending on the choice of combination used. But generally, it will reverse most of the complications that are the direct result of HIV infection [2–4].

Anemia is the most commonly encountered hematologic abnormality, occurring in approximately 30% of patients with asymptomatic HIV and in as many as 75% to 80% of those with clinical AIDS; making it more common than thrombocytopenia or leucopenia in patients with AIDS [5, 6]. Its prevalence is significantly higher among HAART naive patients than those on HAART [7]. Anemia is associated with progression to AIDS [8]**,** shorter survival times [9],and it is a predictor of poorer prognosis for HIV infected patients independent of the CD4 count [10]**.**

Leucopenia and neutropenia and/or lymphopenia often accompany HIV, and their prevalence increases from asymptomatic HIV-infected individuals to individuals with AIDS. Severe uncommon infections such as spontaneous bacterial infections, and rare fungal infections like aspergillosis or mucormycosis may occur in the course of HIV infection due to neutropenia, which may be seen in half of HIV-infected patients. The commonest cause of neutropenia is the result of drugs such as Zidovudine (AZT), the anti-CMV drug ganciclovir, or drugs used to treat cancers and tumors. [11, 12].

Thrombocytopenia is a common finding in individuals infected with HIV, occurring in about 40% of HIV-infected patients and the degree is generally mild to moderate; however, severe reduction of platelet count below 50,000/μL also occurs. With the advent of HAART, thrombocytopenia is more commonly seen in the setting of uncontrolled HIV infection and Hepatitis C co-infection. Thrombocytopenia in HIV infection can be due to either primary HIV-associated thrombocytopenia or secondary thrombocytopenia. Of which PHAT is the most common cause; clinically it resembles classic ITP. [13–15].

Despite the fact that hematologic manifestations of HIV infection are a well-recognized complication of the disease and it increases progression to AIDS; there are no studies done on its magnitude in Ethiopia. Hence this research aims to determine the magnitude and possible associated factors of hematological abnormalities among HIV-infected adult patients in the ART follow-up clinic of Jimma University Specialized Hospital, Jimma, Ethiopia.

## Methods

### Study design

A quantitative cross-sectional study was conducted from July 2012 to September 2012, at Jimma University Specialized Hospital, Jimma, Ethiopia; one of the teaching and referral Hospital in the country.

### Sample size determination, sampling procedure and study subjects

All adult patients diagnosed with HIV and attending the ART follow-up clinic of Jimma University Specialized Hospital were the source population.

The sample size was calculated based on single population formula using a confidence interval (CI) of 95% and previous prevalence of cytopenias being 50% (unknown) among HAART and HAART naïve HIV-infected adult patients. A sample size of 361 was calculated and Quota sampling technique was used to recruit 130 HAART and 231 HAART naïve patients.

The study population was all adult HIV- infected patients who were on follow-up care at Jimma University Specialized Hospital during July 2012 to September 2012. Those adult patients, who have received chemotherapeutic agents (for any malignancy) within 6 months prior to the study, and blood transfusion within 3 months prior to the study, were excluded. Pregnant women and patients unable to give consent were also excluded from the study.

### Data collection instrument and procedures

Structured questionnaires in English were used to collect the data. The questionnaires were constructed with socio-demographic characteristics, clinical data (duration of HIV infection, WHO clinical stage, opportunistic infection, malignancy and other co morbidity status) and medication data (ART status, ART regimen, ART duration and use of CPS).

A total of six health professionals, four ART nurses and an intern as data collectors, and the principal investigator as supervisor were involved in the data collection process of the study.

The study population was separated into HAART and HAART naïve groups. Each client who visited the clinic during the study period was evaluated for eligibility to be included in the study. Those eligible were included in the study consecutively for either of the two groups. The selection and inclusion of patients was continued until the specific number of patients (quota) was obtained for each category.

After patient interview and a detailed review of the medical record, about 4 ml of venous blood was collected by experienced laboratory technologist from each subject for immunologic (CD4 count) and hematologic parameters (CBC) analysis.

### Data quality control

Pretesting of the questionnaires was done in a sample of 20 HIV-infected adult patients attending ALERT Hospital, located in the capital Addis Ababa, Ethiopia, before the actual study begun. The collected data was checked for completeness and internal consistency. Necessary correction of questionnaire was made accordingly after the pre-test. These subjects were not included in the actual study result. Training was given to recruited data collectors. During data collection, the principal investigator ensured quality data collection by supervision, on spot corrective action and recollecting data on 5% of the study population. Each day the principal investigator reviewed all collected data, he checked for completeness and internal consistency and took immediate remedial action accordingly. Each sampled blood was analyzed within two hours using the Cell-Dyn 1800 auto analyzer machine for CBC and the Becton Dickenson(BD) FACS caliber for CD4 count. The performance of these machines was controlled by running quality control samples alongside the study subjects’ sample.

### Operational definitions

The 2011 World Health Organization (WHO) report on Hb concentration level to diagnose anemia was used to define and grade anemia [16]. Accordingly, for males Hb concentration of 13 g/dL or higher was considered non-Anemia(normal) and **anemia** was defined as Hb concentration < 13 g/dL (11.0–12.9 g/dL = mild; 8.0– 10.9 g/ dL moderate, and < 8.0 g/dL = severe), whereas for females Hb concentration of 12 g/dL or higher was considered non-Anemia(normal) and **anemia** was defined as Hb < 12.0 (11.0–11.9 g/ dL = mild, 8.0–10.9 g/ dL = moderate, and < 8.0 g/dL = severe).

Patterns of anemia were classified as **normocytic** (MCV 80 -100 fL), **microcytic** (MCV < 80 fL), **macrocytic** (MCV > 100 fL), **normochromic** (MCH ≥27 pg) and **hypo chromic** (MCH < 27 pg). **MCHC** of < 32.3 g/dl, 32.3-35.9 g/dl and ≥ 36 g/dl were considered low, normal and high respectively.

**Leucocyte count of 4-11** ×  10^3^/μl was considered normal and **leucopenia** was defined as total WBC count < 4 × 10^3^ /μl. Neutrophils constitute 40-70% of the total WBC count and **neutropenia** was defined as absolute neutrophil count < 1000 cells/μl. Lymphocytes constitute 20-50% of the total WBC count and **lymphopenia** was considered when absolute lymphocyte count is < 800 cells/μl. **Platelet** count of 150-450× 10^3^/μl was considered normal and **Thrombocytopenia** was defined as total platelet count < 150 × 10^3^/μl [17].

**Isolated cytopenia**- presence of anemia, thrombocytopenia, or leucopenia.

**Bicytopenia-** presence of any 2 of the following 3: anemia, thrombocytopenia and leucopenia.

**Pancytopenia**- presence of anemia, thrombocytopenia, and leucopenia all together.

A normal **CD4** count in adults ranges from 500 to 1200 cells/mm^3.^

Subjects were classified in to **stage1**, **stage 2**, **stage 3** and **stage 4**, based on WHO clinical staging of HIV disease in adults and adolescents [18].

**HAART** use was defined as receipt of two nucleoside reverse transcriptase inhibitors (NRTI) and one non-nucleoside reverse transcriptase inhibitor (NNRTI) or one protease inhibitor (PI).

### Data processing and analysis

The collected data were categorized, coded, entered onto a computer, cleaned (verified) and analyzed using SPSS Windows version 20.0 software packages for statistical analysis. Descriptive analysis was done to determine the prevalence of anemia, leucopenia, neutropenia, lymphopenia, and thrombocytopenia; and patterns of anemia; and was presented using tables, diagrams and summary measures as appropriate. Before analysis, continuous variables were checked on whether they were normally distributed or not using normal graph curves. Normally distributed Continuous variables were compared with dependent variables using T- test and ANOVA; when ANOVA revealed significant difference further post hoc-multivariate comparison were done. Categorical variables were compared with dependent variables using chi- square test and fisher exact test when appropriate. When association was found the odds ratio was used to measure the strength of association. Finally, for variables which had statistically significant association with dependent variable multiple logistic regression analysis were done to come up with the independent predictors of outcome variables (dependent variable). All tests were two tailed and statistical significance was considered at *p* < 0.05 with 95%CI.

## Results

### Socio-demographic characteristics

Three hundred sixty-one HIV infected individuals were involved in this study, made up of 149(41.3%) males and 212(58.7%) females (Table 1). The overall mean age for the study population was 34.7 ± 10.1 years (range 16-70). The mean age of 32.1 ± 9.37 and 38.4 ± 10.04 years was obtained for females and males, respectively, with a minimum of 16 and 17, and a maximum of 70 and 68 years for females and males, respectively (Table 2). Majority of the study subjects (87.8%) were living in urban areas and more than three fourth (77.5%, *n* = 280) of the subjects had at least primary school education or more. Nearly half (49.6%, *n* = 179) of the participants were married, 18.8% single, 21.6% divorced and 10% were widows or widowers. 22.7% of the individuals were dependent and 54% earned monthly personal income below 500 birr ($ 27.78) (Table 1).

**Table 1:** Distribution of socio-demographic characteristics of the study subjects, at JUSH from July-September 2012

Variables	On HAART N (%)	HAART naive N (%)	Total N (%)
Age in years			
16-26	17(13.1)	61(26.4)	78(21.6)
27-37	56(43.1)	107(46.3)	163(45.2)
38-48	40(30.8)	46(19.9)	86(23.8)
49-59	15(11.5)	7(3.0)	22(6.1)
60-70	2(1.5)	10(4.3)	12(3.3)
Total	130(100)	231(100)	361(100)
Mean age ± SD	33.5 ± 10.3	36.8 ± 9.5	
Sex			
Male	50(38.5)	99(42.9)	149(41.3)
Female	80(61.5)	132(57.1)	212(58.7)
Marital status			
Married	57(43.8)	122(52.8)	179(49.6)
Single	17(13.1)	51(22.1)	68(18.8)
Divorced	34(26.2)	44(19.0)	78(21.6)
Widowed	22(16.9)	14(6.1)	36(10.0)
Monthly personal income (Birr)			
< 500	84(64.6)	111(48.1)	195(54.0)
500-1000	21(16.2)	91(39.4)	112(31.0)
1001-1500	13(10.0)	24(10.4)	37(10.2)
> 1500	12(9.2)	5(2.2)	17(4.7)
Total	130(100)	231(100)	361(100)

**Table 2:** Clinical and hematological parameter distribution of the study subjects, at JUSH from July-September 2012

Variables	Female		Male		HAART patients		HAART naïve		Total	
	Min-Max	Mean ± SD	Min-Max	Mean ± SD	Min-Max	Mean ± SD	Min-Max	Mean ± SD	Min-Max	Mean ± SD
Age	16-70	32.1 ± 9.38	17-68	38.4 ± 10.04	20-67	36.9 ± 9.5	16-70	33.5 ± 10.3	16-70	34.7 ± 10
HIV dur. (months)	1-117	31 ± 27.6	1-96	29 ± 24.3	1-117	50.6 ± 25.7	1-90	18.9 ± 18.7	1-117	30 ± 26.3
ART dur. (months)	1-96	41 ± 25.8	1-76	36 ± 21.9	1-96	39 ± 24.5	–	–	1-96	39.11 ± 24.46
CD4 count	5-1377	406 ± 232.6	36-1421	343 ± 199.07	5-1314	429.2 ± 337.5	7.6-16.9	352 ± 207	5-1421	380 ± 221
Hemoglobin	4.3-15.6	11.9 ± 1.65	7.8-18.6	12.6 ± 2.1	4.3-18.6	12.6 ± 1.85	2.4-13	11.9 ± 1.8	4.3-18.6	12.2 ± 1.9
WBC × 10^3^	1.8-13.0	6.007 ± 1.92	2.3-11.6	6.069 ± 1.78	1.8-12.5	6.28 ± 2	1.2-9.88	5.89 ± 1.76	1.8-13	6.03 ± 1.86
ANC × 10^3^	1.062-9.88	3.549 ± 1.56	.920-8.424	3.530 ± 1.39	0.92-9.5	3.76 ± 1.7	0.35-5.05	3.42 ± 1.3	0.92-9.88	3.54 ± 1.48
TLC × 10^3^	0.349-5.05	1.854 ± 0.76	.543-4.325	1.854 ± 0.706	0.57-4.3	1.9 ± 0.7	13-987	1.82 ± 0.75	0.349-5.05	1.85 ± 0.74
Platelet count × 10^3^	62.0-987.0	296.769 ± 135.22	13-890	276.953 ± 149.89	73-750	290.3 ± 111.5	21-1421	287.6 ± 156.2	13-987	288.59 ± 141.59

### Base line clinical profile

The mean duration since the diagnosis of HIV for the study subjects was 30.3 ± 26.3 months (range 1-117). The mean duration for females and males was 31 ± 27.6 and 29 ± 24.3 months, respectively, with a minimum of 1 month for both and a maximum of 117 and 96 months for females and males, respectively (Table 2). According to WHO clinical staging, about two third (64%, *n* = 231) of the study subjects were in stage I and II, while 36% (*n* = 130) of the individuals had advanced clinical stage (stage III and IV) (Table 3).

**Table 3:** Clinical and immunological characteristics of the study subjects, at JUSH from July-September 2012

Variables	Females N (%)	Males N (%)	Total N (%)
WHO clinical stage			
Stage I	56(62.2)	34(37.8)	90(24.9)
Stage II	80(56.7)	61(43.3)	141(39.1)
Stage III	66(64.7)	36(35.3)	102(28.3)
Stage IV	10(35.7)	18(64.3)	28(7.8)
Opportunistic infections			
TB	12(46.2)	14(53.8)	26(7.2)
Chronic GE	6(50)	6(50)	12(3.3)
Pneumonia	1(20)	4(80)	5(1.4)
Candidiasis	4(80)	1(20)	5(1.4)
No Total	189(60.4) 212(58.7)	124(39.6) 149(41.3)	313(86.7) 361(100)
Co- morbidities			
HTN	2(100)	0(0)	2(0.5)
DM	0(0)	2(100)	2(0.5)
CLD	2(100)	0(0)	2(0.5)
No Total	208(58.6) 212(58.7)	147(41.4) 149(41.3)	355(98.3) 361(100)
Immunologic stage			
CD4 ≥ 500	53(69.7)	23(30.3)	76(21.1)
CD4 200-499	127(57.7)	93(42.3)	220(60.9)
CD4 < 200	32(49.2)	33(50.8)	65(18.0)

13.3% (*n* = 48) of the individuals had history of opportunistic infection (OI) at the time of the study, the commonest being tuberculosis (TB) seen in 54.2% (*n* = 26) of cases, followed by chronic GE, pneumonia and candidiasis (oral± esophageal) seen in 25% (*n* = 12), 10.4% (*n* = 5) and 10.4% (n = 5) of cases, respectively (Table 3).

The mean CD4 lymphocyte count for the study population was 380 ± 221 (range 5-1421). Mean CD4 count of 406 ± 232.6 and 343 ± 199 cells/μl was obtained for females and males, respectively, with a minimum of 5 and 36 and a maximum of 1377 and 1421 cells/μl for females and males respectively (Table 2). The majority of the study subjects (60.9%) had a CD4 count of 200-499 cells/μl; whereas 21.1% and 18% of the subjects had CD4 lymphocyte count of ≥500 cells/μl and < 200 cells/μl, respectively (Table 3).

Of the 361 individuals about two third (64%, *n* = 231) were HAART naive and 36% (*n* = 130) were taking HAART. 37.7% (*n* = 80) of female and 33.6% (*n* = 50) of male study subjects were on HAART (Table 3). Mean age of 33.5 ± 10.3 and 36.8 ± 9.5 years was obtained for HAART naïve and HAART patients respectively (*p* = 0.002) (Table 2). The mean CD4 count of HAART patients (429 ± 237) was 1.2 times higher than that of their HAART naïve counterparts (352 ± 207) (*p* = 0.01). 40% of HAART patients (*n* = 52) were taking TDF based first line regimen, while 39.2% (*n* = 51) and 20% (*n* = 26) of individuals were taking AZT based and D4T based first line regimen, respectively, whereas only one individual (0.8%) was taking TDF based second line regimen. The overall mean duration since the initiation of HAART was 39 ± 24.5 months (range 1-96 months), it was 41 ± 25.8 and 36 ± 21.9 months for females and males respectively (Table 2).

60.9%(*n* = 220) of the study subjects were taking co-trimoxazole prophylaxis therapy, 7.5%(*n* = 27) were taking INH prophylaxis, 7.2%(n = 26) were taking anti TB treatment and another 7.5%(n = 27) were taking different drugs including fluconazole, antibiotics and vitamins.

### Mean cell count values and prevalence of cytopenias

#### Hemoglobin concentration and anemia prevalence, severity and pattern

Anemia was the most common cytopenia in this study; seen in 186 (51.5%) cases (Table 4). The prevalence of anemia was 47.2% (100/212) in female patients and 57.7% (86/149) in male patients (*p* = 0.062) (Table 4), it was 56.3%(130/231) and 43.1%(56/130) for HAART naïve and HAART patients, respectively, (*p* = 0.021) (Table 5). Anemia prevalence was 88.9% for patients with CD4 count < 50 cells/μl and 85.7% for CD4 count 50-99 cells/μl, 81% for CD4 count 100-199 cells/μl, 65.3% for CD4 count 200-349 cells/μl, 32.7% for CD4 count 350-500 cells/μl and 28.6% for CD4 count > 500 cells/μl (*p* = 0.000) (Table 6). We found 71.4% prevalence of anemia for WHO clinical stage IV patients and 62.7% for stage III, 57.4% for stage II and 23.3% for stage I patients (*p* = 0.000) (Table 7). There was no difference between the prevalence of anemia in relation to ART regimen (AZT vs non-AZT based) (*p* = .0.58) (data not shown).

**Table 4:** Frequency distribution of cytopenia in the study subjects, at JUSH from July-September 2012 (*n* = 361)

Cytopenia	Female (*n* = 212) N (%)	Male (*n* = 149) N (%)	Total (361) N (%)
Anemia	100(53.8)	86(57.7)	186(51.5)
Leucopenia	28(13.2)	19(12.8)	47(13)
Thrombocytopenia	18(8.5)	22(14.80	40(11.1)
Lymphopenia	13(6.1)	5(3.4)	18(5)
Neutropenia	0(0)	1(0.7)	1(0.3)
Isolated cytopenia	100(47.2)	83(55.7)	183(50.7)
Bicytopenia	9(4.2)	13(8.70	22(6.1)

**Table 5:** Distribution of categories of hematologic values of the study subjects, at JUSH from July-September 2012

Variables	On HAART N (%)	HAART naïve N (%)	Total N (%)	*P*-value
Hemoglobin (g/dl)				
Not anemic	74(56.9)	101(43.7)	175(48.5)	0.021
Anemic	56(43.1)	130(56.3)	186(51.5)	
Total	130(100)	231(100)	361(100)	
Anemia severity				
Grade 1	50(89.3)	98(75.4)	148(79.6)	
Grade 2	4(7.1)	32(24.6)	36(19.3)	
Grade 3	2(3.6)	0(0.0)	2(1.1)	
Total	56(100)	130(100)	186(100)	
WBC (cells/μl)				
≥4000	114(87.7)	200(86.6)	314(87.0)	0.890
< 4000	16(12.3)	31(13.4)	47(13.0)	
Total	130(100)	231(100)	361(100)	
ANC (cells/μl)				
≥1000	129(99.2)	231(100.0)	360(99.7)	
< 1000	1(0.8)	0(0.0)	1(0.3)	
Total	130(100)	231(100)	361(100)	
TLC (cells/μl)				
≥800	126(96.9)	217(93.9)	343(95.0)	0.318
< 800	4(3.1)	14(6.1)	18(5.0)	
Total	130(100)	231(100)	361(100)	
Platelet count (cells/μl)				
≥150 × 10^3^	121(93.1)	200(86.6)	321(88.9)	0.087
< 150 × 10^3^	9(6.9)	31(13.4)	40(11.1)	
Total	130(100)	231(100)	360(100)

**Table 6:** Association of immunologic stages with cytopenias in the study subjects, at JUSH from July-September 2012

Immunologic stage	Total N (%)	Anemia N (%)	P-value	Leucopenia N (%)	*P*-value	Thrombocytopenia N (%)	P-value	Lymphopenia N (%)	*P*-value
> 500	77 (21.3)	22(28.6)	0.000	3(3.9)	0.000	5(6.5)	0.261	0(0)	0.000
350-500	101 (28)	33(32.7)		6(5.9)		10(9.9)		0(0)	
200-349	118 (32.7)	77(65.3)		16(13.6)		13(11)		5(4.20	
100-199	42 (11.6)	34(81.00		14(33.3)		9(21.4)		5(11.9)	
50-99	14 (3.9)	12(85.7)		4(28.6)		2(14.3)		6(42.9)	
< 50	9 (2.5)	8(88.9)		4(44.4)		1(11.1)		2(22.2)	
Total	361(100)	186(51.5)		47(13)		40(11.1)		18(5)

**Table 7:** Association of clinical stage with cytopenia in the study subjects, at JUSH from July-September 2012

Clinical stage	Total N (%)	Anemia N (%)	P-value	Leucopenia N (%)	P-value	Thrombocytopenia N (%)	*P*-value	Lymphopenia N (%)	*P*-value
Stage I	90(24.9)	21(23.3)	0.000	9(10)	0.132	9(10)	0.982	2(2.2)	0.138
Stage II	141(39.1)	81(57.4)		15(10.6)		16(11.3)		5(3.5)	
Stage III	102(28.3)	64(62.7)		16(15.7)		12(11.8)		9(8.8)	
Stage IV	28(7.8)	20(71.4)		7(25)		3(10.7)		2(7.1)	
Total	361(100)	186(51.5)		47(13)		40(11.1)		18(5)	

Most of the anemic patients, 79.6%(*n* = 148), had mild degree of anemia (Hb ≥ 11 g/dl), while 19.3%(*n* = 36) had moderately severe anemia (Hb 8-10.9 g/dl) and two individuals (1.1%) had severe anemia (Hb < 8 g/dl) (Table 5). Moderately severe anemia was seen in 35.2% and 13.6% of patients with CD4 count < 200cells /μl and CD4 count ≥200cells /μl, respectively, (*p* = 0.001); in 9.5%, 12.3%, 28.1% and 35% of patients with WHO clinical stage I, II, III and IV, respectively, (*p* = 0.007) (data not shown); in 7.1% and 24.6% of HAART and HAART naïve patients, respectively (*p* = 0.017) (Table 5). Whereas, there was no difference between the severity of anemia in relation to gender.

**Fig. 1:**
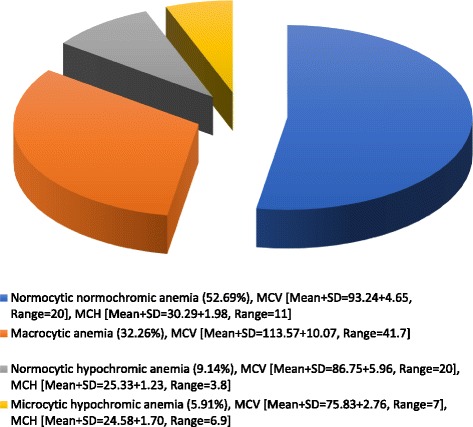
Pie-Chart - pattern of anemia among the study subjects at JUSH, July-September 2012 (*n* = 186)

The most common pattern of anemia was normocytic normochromic in 98 (52.7%) cases followed by macrocytic anemia in 60 (32.2%) cases, normocytic hypo chromic anemia in 17 (9.1%) cases and microcytic hypo chromic anemia in 11(5.9%) cases (Fig. 1). Normocytic normochromic, normocytic hypo chromic, microcytic hypo chromic and macrocytic anemia were seen in 60.8%, 12.3%, 8.5% and 18.5% and 33.9%, 1.8%, 0% and 64.3% of HAART naïve and HAART patients, respectively, (*p* = 0.000). Whereas there was no difference between the pattern of anemia in relation to gender, WHO clinical stage and immunologic stage (data not shown).

The mean ± standard deviation (SD) hemoglobin concentration for the study population was 12.2 ± 1.86 (range 4.3-18.6) (Table 2), it was 11.9 ± 1.65 g/dl and 12.6 ± 1.65 g/dl for female and male patients respectively (*p* = 0.000) (Table 2), 10.9 ± 1.9 g/dl and 12.47 ± 1.7 g/dl for CD4 count < 200 cells/μl and ≥ 200 cells/μl, respectively (p = 0.000) (data not shown), 12.6 ± 1.84 and 11.9 ± 1.83 for HAART and HAART naïve patients, respectively (Table 2) (*p* = 0.002) and it was 12.5 ± 1.77 and 11.6 ± 1.87 for patients with early and advanced WHO clinical stages, respectively (*p* = 0.000). There was no difference between the mean hemoglobin concentration in relation to ART regimen (data not shown).

#### Mean WBC count and prevalence of leucopenia

Leucopenia (WBC < 4000/μL) was found in13%(*n* = 47) of the participants, the prevalence was 13.2% (28) in female patients and 12.8% (19) in male patients (Table 4), (*p* = 1.000). Leucopenia was found in 3.9%, 5.9%, 13.6%, 33.3%, 28.6% and 44.4% of patients with CD4 count> 500 cells/μl, 350-500 cells/μl, 200-349 cells/μl, 100-199 cells/μl, 50-99 cells/μl and < 50 cells/μl respectively (*p* = 0.000) (Table 6). Whereas there was no difference between the prevalence of leucopenia in relation to WHO clinical stages (Table 7), ART status (Table 5) and ART regimen (data not shown).

The mean ± standard deviation (SD) WBC count for the study population was 6.03 ± 1.86 × 10^3^ (range 1.8-13) (Table 2), it was 5.11 ± 1.88 and 6.24 ± 1.8 × 10^3^ for patients in the CD4 count group < 200 and ≥ 200/μl, respectively, (*p* = 0.000) (data not shown). Otherwise there was no difference between the mean WBC count in relation to gender, WHO clinical stages, ART status and ART regimen (data not shown).

#### Mean platelet count and prevalence of thrombocytopenia

Thrombocytopenia (platelet< 150,000/μL) was found in 11.1%(*n* = 40) of the study subjects, the prevalence was 8.5%(18/212) and 14.8%(22/149) for female and male patients respectively (Table 4), (*p* = 0.089); 2%(1/51) and 10.1%(8/79) for AZT and non AZT based HAART patients, respectively, (*p* = 0.579) (data not shown).There was no difference between the prevalence of thrombocytopenia in relation to immunologic stage (CD4 category) (Table 6), WHO clinical stages (Table 7) and ART status (Table 5).

The mean ± standard deviation (SD) platelet count for the study population was 288.6 ± 141.6 × 10^3^ (range 13-987) (Table 2), it was 255.3 ± 135.7 and 295.9 ± 142.02 × 10^3^ for patients in the CD4 count group < 200 and ≥ 200/μl, respectively, (*p* = 0.036); 303.7 ± 119.2 and 281.7 ± 106.2 × 10^3^ for AZT and non-AZT based HAART, respectively, (*p* = 0.273). There was no difference between the mean platelet count in relation to gender, WHO clinical stages, ART status and ART regimen (data not shown).

#### Mean TLC and prevalence of lymphopenia

Lymphopenia (TLC < 800/μL) was found in 5%(*n* = 18) of the participants, the prevalence was 6.1%(13/212) and 3.4%(5/149) for female and male patients, respectively (Table 4), (*p* = 0.343). The prevalence of lymphopenia was 0%, 0%, 4.2%, 11.9%, 42.9% and 22.2% for patients with CD4 count > 500, 350-500, 200-349, 100-199, 50-99 and < 50 cells/μl, respectively, (*p* = 0.000) (Table 6). Otherwise there was no difference between the prevalence of lymphopenia in relation to WHO clinical stages (Table 7), ART status (Table 5) and ART regimens (data not shown).

The mean ± standard deviation (SD) TLC for the study population was 1.85 ± 0.735 × 10^3^ (range 0.349-5.05) (Table 2), it was 1.39 ± 0.66 and 1.96 ± 0.7 × 10^3^ for patients in the CD4 count group < 200 and ≥ 200/μl, respectively, (*p* = 0.000); whereas there was no difference between the mean TLC in relation to gender, WHO clinical stages, ART status and ART regimens (data not shown).

#### Mean ANC and prevalence of neutropenia, isolated cytopenia and bicytopenia

Neutropenia (ANC < 1000/μL) was present in only one individual (0.3%) and isolated cytopenia and bicytopenia were found in 50.7%(*n* = 183) and 6.1%(*n* = 22) of the participants respectively, and there was no one with Pancytopenia (Table 4).

The mean ± standard deviation (SD) ANC for the study population was 3.54 ± 1.49 × 10^3^ (range 0.92-9.88) (Table 2), it was 3.42 ± 1.3 and 3.76 ± 1.74 × 10^3^ for HAART naïve and HAART patients (Table 2), respectively, (*p* = 0.037) and 3.2 ± 1.46 and 3.6 ± 1.5 × 10^3^ for patients in the CD4 count group < 200 and ≥ 200/μl, respectively, (*p* = 0.031) (data not shown); whereas there was no difference between the mean ANC in relation to gender, WHO clinical stages and ART regimens (data not shown).

#### Risk factors for cytopenias

A univariate analysis showed that many risk factors were associated with prevalent anemia. Risk factors associated with increased risk of anemia were: Advanced WHO clinical stage OR 2.3(IC 95% 1.48-3.59; *p* = 0.000), presence of opportunistic infection OR 10.2(IC 95% 3.94-26.5; *p* = 0.000), lack of HAART OR 1.7(IC 95% 1.1-2.6; *p* = 0.021), use of co-trimoxazole prophylaxis therapy OR 1.99(IC 95% 1.29-3.05; *p* = 0.002), CD4 count < 200 cells/μl OR 6.1(IC 95% 3.1-12.1; *p* = 0.000), WBC count < 4000/μl OR 2.78(IC 95% 1.41-5.48; *p* = 0.004) and TLC < 800 cells/μl OR 17.5(IC 95% 2.3-132.98; *p* = 0.000). Presence of opportunistic infection, use of co-trimoxazole prophylaxis therapy and CD4 count < 200 cells/μl were associated with an increased risk of anemia in the multivariate analysis with OR 5.72(IC 95% 2.05-15.97; *p* = 0.001), OR 1.65(IC 95% 1.02-2.67; *p* = 0.040) and OR 3.34(IC 95% 1.57-7.1; *p* = 0.002) respectively (Table 8).

**Table 8:** Factors associated with anemia in the study subjects, at JUSH from July-September 2012

Variables	Anemia present *n* = 186 N (%)	No anemia *n* = 175 N (%)	COR (95% CI) *p*-value	AOR (95% CI) *p*-value
Clinical stage				
Advanced (stage III and IV) Early (stage I and II)	84(45.2) 102(54.8)	46(26.3) 129(73.7)	2.3(1.48-3.6) 0.000	1.59(0.93-2.72) 0.089
Opportunistic infection				
Present Absent	43(23.1) 143(76.9)	5(2.9) 170(97.1)	10.2(3.94-26.5) 0.000	5.72(2.05-15.92) 0.001
HAART status				
HAART naïve On HAART	130(69.9) 56(30.1)	101(57.7) 74(42.3)	1.7(1.1-2.6) 0.021	1.53(0.91-2.56) 0.109
Use of CPT				
On CPT Not on CPT	128(68.8) 58(31.2)	92(52.6) 83(47.4)	1.99(1.29-3.05) 0.002	1.65(1.02-2.67) 0.04
CD4 count				
1 < 200 ≥200	54(29) 132(71)	11(6.3) 164(93.7)	6.1(3.1-12.1) 0.000	3.34(1.57-7.1) 0.002
WBC count				
< 4000 ≥4000	34(18.3) 152(81.7)	13(7.4) 162(92.6)	2.78(1.41-5.48) 0.004	1.44(0.66-3.14) 0.356
TLC				
< 800 ≥800	17(9.1) 169(90.9)	1(0.6) 174(99.4)	17.5(2.3-132.98) 0.000	7.26(0.867-60.74)

A univariate analysis showed that use of co-trimoxazole prophylaxis therapy, CD4 count < 200cells /μl, presence of anemia and presence of lymphopenia were associated with increased risk of leucopenia with OR 3.06(IC 95% 1.43-6.55; *p* = 0.005), OR 5.54(IC 95% 2.87-10.7; *p* = 0.000), OR 2.78(IC 95% 1.41-5.48; *p* = 0.004) and OR 6.23(IC 95% 2.32-16.74; *p* = 0.000), respectively. In the multivariate analysis only CD4 count < 200 cells/μl and use of co-trimoxazole prophylaxis therapy were associated with increased risk of leucopenia with OR 3.34(IC 95% 1.59-7.02; *p* = 0.001) and OR 2.34(IC 95% 1.05-5.19; *p* = 0.036) respectively (Table 9).

**Table 9:** Factors associated with prevalent leucopenia in the study subjects, at JUSH from July-September 2012

Variables	Leucopenia present *n* = 47 N (%)	No leucopenia *n* = 314 N (%)	COR (95% CI) *p*-value	AOR (95% CI) *p*-value
Use of CPT				
On CPT Not on CPT	38(80.9) 9(19.1)	182(58) 132(42)	3.06(1.43-6.55) 0.005	2.34(1.05-5.19) 0.036
CD4 count				
< 200 ≥200	22(46.8) 25(53.2)	43(13.7) 271(86.3)	5.54(2.87-10.7) 0.000	3.34(1.59-7.02) 0.001
Anemia status				
Present Absent	34(72.3) 13(27.7)	152(48.4) 162(51.6)	2.78(1.41-5.48) 0.004	1.6(0.76-3.34) 0.211
TLC				
< 800 ≥800	8(17) 39(83)	10(3.2) 304(96.8)	6.23(2.32-16.74	2.78(0.908-8.54) 0.073

In a univariate analysis, there wasn’t any risk factor associated with prevalence of thrombocytopenia. It was found in 6.5%, 9.9%, 11%, 21.4%, 14.3% and 11.1% of patients with CD4 count > 500 cells/μl, 350-500 cells/μl, 200-349 cells/μl, 100-199 cells/μl, 50-99 cells/μl and < 50 cells/μl respectively (*p* = 0.261) (Table 6). Although statistically not significant HAART naïve patients were more than 2 times at risk of having thrombocytopenia compared to those on HAART (*p* = 0.087) (Table 5). Patients on non-AZT based HAART were more than 5 times at risk of having thrombocytopenia compared to those on AZT based HAART, however it wasn’t statistically significant (*p* = 0.151) (data not shown).

A univariate analysis showed that many risk factors were associated with prevalence of lymphopenia. Risk factors associated with increased risk of lymphopenia were: duration since the diagnosis of HIV ≤ 6 months OR 12.3(IC 95% 3.9-38.4; *p* = 0.000), duration since the initiation of HAART ≤6 months OR 26.07(IC 95% 2.52-269.3; *p* = 0.006), CD4 count < 200 cells/μl OR 14.5(IC 95% 4.97-42.53), presence of anemia OR 17.5(IC 95% 2.3-132.98; p = 0.000), presence of leucopenia OR 6.23(IC 95% 2.32-16.74; p = 0.000) and presence of opportunistic infection OR 3.58(IC 95% 1.27-10.05; *p* = 0.021). In the multivariate analysis, none of them were associated with increased risk of lymphopenia (data not shown).

## Discussion

Our study revealed that 51.5% of the study population was anemic, a value that agrees with the Benin city and India studies [7, 19]. In contrast to other studies, there were no gender difference in the prevalence of anemia in this study, women (47.2%) and men (57.7%) (*p* = 0.062) [20]. Also, we did not observe any difference in the prevalence of microcytic anemia among women (6%) and men (5.8%) (*p* = 0.918). These indicate that iron deficiency was not the main cause of anemia in the study population.

This study confirms that anemia is directly related with the degree of immunosuppression, a finding that agrees with those found by others [20–22]; the lower the CD4 count the higher the prevalence of anemia.

We also proved that anemia is associated with advanced WHO clinical stage, a finding that is in accordance with the literature [18, 20, 22]; the advanced the clinical stage the higher the prevalence of anemia.

In accordance with the Benin city and Rwanda studies [7, 22], we did find a higher prevalence of anemia in HAART naïve patients compared to patients on HAART. We did not observe any significant difference between the hemoglobin level in relation to ART regimens (AZT based HAART vs non-AZT based HAART); a finding that differs from those observed by others who have observed a higher prevalence of anemia in patients taking AZT based HAART [20]. The possible explanation could be that, in contrast to the pre-HAART era, the risk of anemia with AZT therapy has been reduced with the advent of HAART [23].

Our study revealed that use of co-trimoxazole prophylaxis therapy is positively associated with prevalence of anemia, a finding in sharp contrast with that found by others, who have found a negative association between use of CPT and prevalence of anemia [20]. One possible explanation could be that trimethoprim is a weak inhibitor of dihydrofolate reductase and in high doses, it has been implicated in Megaloblastic Pancytopenia, particularly in patients who are not on folate supplementation like our patients [24]. However, the usage of co trimoxazole therapy wasn’t different between macrocytic (73.3%) and other patterns of anemia (66.6%) (*p* = 0.816).

Moreover, this study found that, presence of an opportunistic infection at the time of the study was associated with an increased prevalence of anemia, irrespective of clinical stage and degree of immunosuppression, which could perhaps be explained by anemia related to secondary infections.

The study found normocytic normochromic anemia as the most common pattern of anemia (52.7%); a finding that is in agreement with the literature [25, 26]. Macrocytic RBC morphology was seen in 32.2% of the anemic subjects, a finding with a value higher than others (9%) [25]. Possible explanation for this could be higher rate of co trimoxazole usage in our patients, which as already mentioned above is associated with Megaloblastic anemia in patients who lack folate supplementation.

The prevalence of leucopenia in our patients, defined as WBC count less than 4000/μl, was 13%, a value somewhat lower than those found by other studies, which were done on HAART naive patients [21, 25]. The possible explanation for this could be the presence of HAART patients in our study which, to a certain degree, lowered the prevalence.

The study confirmed that leucopenia is directly related with the degree of immunosuppression, a finding that agrees with the literature [21]. Our study also revealed that use of co-trimoxazole prophylaxis therapy is positively associated with prevalence of leucopenia, a finding not addressed by others. This could be due to the megaloblastic side effect of co trimoxazole therapy in the absence of folate supplementation.

The prevalence of thrombocytopenia (platelet count less than 150 × 10^3^) in our patients was 11.1%, a value somewhat similar to that found by others [19]. This study confirmed that prevalence of thrombocytopenia is not associated with neither the degree of immunosuppression nor with the clinical stage of HIV, a finding that is in accordance with the literature [18, 21]. Most importantly, this study also showed a higher prevalence of thrombocytopenia among patients on non-AZT based HAART regimen as compared to those on AZT based HAART regimen. The possible explanation could be that AZT can rapidly increase platelet count in patients with HIV related thrombocytopenia [27, 28].

The prevalence of lymphopenia in our patients, defined as TLC less than 800 cells/μl, was 5%, a value lower than those found by a study undertaken on HAART naive patients [29]. This could be due to the presence of HAART patients in our study which, to a certain degree, lowered the prevalence. As expected, we found that patients with CD4 count less than 200cells/μl presented with a significantly increased prevalence of lymphopenia. Our study revealed that lymphopenia is inversely related with the duration of HIV infection, higher prevalence observed in a group of patients diagnosed in the prior 6 months. This might be due to the rapid viral replication and associated lymphocyte cell death occurring in the acute phase of HIV infection. The study also revealed that prevalence of lymphopenia is inversely related with the duration of HAART treatment, lower prevalence observed in a group of patients with HAART duration greater than 6 months. This could be due to the hematologic recovery that occurs after 6 months of HAART treatment [29].

Neutropenia, defined as ANC less than 1000/μl, was found only in one individual, a finding that agrees with the Indian study which found no patient with neutropenia [26]. The mean Neutrophil count was lower in HAART naïve and immunologic AIDS patients, a finding that is in accordance with the literature [27, 28, 30].

In summary, the hematologic abnormalities that we have observed in our patients could be the direct result of the HIV infection itself; nutritional deficiencies; manifestations of secondary infections and neoplasms, or side effects of therapy. Cytopenias, particularly anemia, could be associated with progression to AIDS, shorter survival times, and a predictor of poorer prognosis in our patients.

## Conclusion and recommendation

### Conclusion

In conclusion in this study anemia, leucopenia, thrombocytopenia and lymphopenia were common among the study subjects in both HAART naïve and HAART patients. Of the cytopenias, anemia was the most common manifestation and the most frequent patterns were normocytic normochromic and macrocytic forms, whereas neutropenia was rare.

Prevalence of anemia, leucopenia and lymphopenia correlates with disease progression. Thrombocytopenia occurs independent of disease progression. The present study also showed that use of co-trimoxazole prophylaxis therapy was independently associated with an increased risk of anemia and leucopenia; a finding that needs to be strengthened by further study.

#### Recommendation

Based on these findings it is recommended that physicians giving care for HIV infected adults should routinely investigate and treat hematologic abnormalities in both HAART and HAART naïve patients to reduce the morbidity and mortality of the patients.

Additionally, co trimoxazole prophylaxis therapy should be avoided in HIV-infected adult patients who have folic acid deficiency or who are pregnant, and instead the other alternatives for PCP prophylaxis can be used. And in the rest group of HIV patients co trimoxazole prophylaxis therapy should only be used if the potential benefit outweighs the possible hematologic risk (patients with CD4 count< 200 cells/μl, prior bout of PCP).

However, large scale and longitudinal studies, giving emphasis on the association of co trimoxazole prophylaxis therapy and cytopenia, are recommended to strengthen and explore the problem in depth.

## Abbreviation

AIDS: Acquired Immuno deficiency syndrome
ANC: Absolute Neutrophil count
ANOVA: One-way analysis of variance
ART: Anti-retroviral therapy
AZT: Zidovudine
CART: Combined Anti-retroviral therapy
CBC: Complete blood count
CDC: Center for disease control
CMV: Cytomegalovirus
CPT: Cotrimoxazole prophylaxis therapy
Fl: Femto liter
GCSF: Granulocyte colony stimulating factor
HAART: Highly active antiretroviral therapy
Hb: Hemoglobin
HIV: Human immunodeficiency virus
ITP: Idiopathic thrombocytopenic purpura
JUSH: Jimma university specialized hospital
MCH: Mean Corpuscular hemoglobin
MCHC: Mean cell hemoglobin concentration
MCV: Mean Corpuscular Volume
NNRTI: Non- Nucleoside reverse transcriptase inhibitor
NRTI: Nucleoside reverse transcriptase inhibitor
PCV: Packed cell volume
Pg: Pico gram
PHAT: Primary HIV associated thrombocytopenia
PI: Protease inhibitor
PLWHA: Patient living with HIV/AIDS
RBC: Red blood cell
SPSS: Statistical Package for Social Science
TB: Tuberculosis
TLC: Total lymphocyte count
TTP-HUS: Thrombotic thrombocytopenic purpura- hemolytic uremic syndrome
WBC: White blood cell count
WHO: World Health Organization
